# Effects of Topically Applied Vitamin D during Corneal Wound Healing

**DOI:** 10.1371/journal.pone.0152889

**Published:** 2016-04-01

**Authors:** Rose Y. Reins, Samuel D. Hanlon, Sri Magadi, Alison M. McDermott

**Affiliations:** College of Optometry, University of Houston, Houston, Texas, United States of America; Cedars-Sinai Medical Center; UCLA School of Medicine, UNITED STATES

## Abstract

Vitamin D is an important regulator of immune function and largely acts to dampen chronic inflammatory events in a variety of tissues. There is also accumulating evidence that vitamin D acts to enhance initial inflammation, beneficial during both infection and wound healing, and then promotes resolution and prevention of chronic, damaging inflammation. The current study examines the effect of topical vitamin D in a mouse of model of corneal epithelial wound healing, where acute inflammation is necessary for efficient wound closure. At 12 and 18 hours post-wounding, vitamin D treatment significantly delayed wound closure by ~17% and increased infiltration of neutrophils into the central cornea. Basal epithelial cell division, corneal nerve density, and levels of VEGF, TGFβ, IL-1β, and TNFα were unchanged. However, vitamin D increased the production of the anti-microbial peptide CRAMP 12 hours after wounding. These data suggest a possible role for vitamin D in modulating corneal wound healing and have important implications for therapeutic use of vitamin D at the ocular surface.

## Introduction

Vitamin D is a pleiotropic molecule that has widespread effects not only on calcium homeostasis, but also cellular differentiation, proliferation, and immune responsiveness [1–4]. 1,25 dihydroxyvitamin D (1,25D_3_), the active form of vitamin D, has been widely studied for its immunomodulatory properties and is known to suppress inflammation in a variety of tissues, largely through its influence on antigen presenting cell differentiation, lymphocyte proliferation, innate immune receptor signaling, and cytokine and chemokine expression [3,5,6]. In addition to its beneficial effects during inflammatory events, vitamin D also induces the expression of antimicrobial peptides [7–9], potentially providing enhanced protection during infection and wound healing.

The cornea is the transparent tissue at the front of the eye that serves both to refract light back onto the retina and to protect the underlying tissues from damage. Following a corneal epithelial abrasion, there is a well-characterized local inflammatory response that is necessary for efficient wound healing and re-epithelialization to help rapidly restore optimal vision [10–13]. This process of corneal wound healing involves interactions between epithelial cells, stromal keratocytes, leukocytes, platelets, and nerves which are mediated by growth factors, cytokines, and adhesion molecules [14]. After epithelial debridement, there is a coordinated response between all of these components to ensure efficient wound closure. Basal epithelial cells migrate into the wounded area and undergo division to re-epithelialize and then re-stratify the injured area. Inflammatory signals from the epithelium induce keratocyte death and injury triggers the infiltration of immune cells into the cornea from the limbal vessels [14–16]. This infiltration is necessary for proper wound healing, as defects in neutrophil trafficking result in delayed re-epithelialization [10–12,17]. In addition to a loss of neutrophil infiltration, too much accumulation also results in delayed wound closure [13,18], demonstrating the delicate balance of inflammatory events needed during corneal healing. Migration of other immune cells, including dendritic cells, Natural Killer (NK) cells, and γδ T lymphocytes also infiltrate into the cornea and influence recovery [12,13,19].

Corneal nerves also contribute to the wound healing process [20]. The cornea is one of the most densely innervated tissues in the body, serving both to protect the cornea from damage and to provide trophic factors necessary for corneal health and normal maintenance [21–24]. Corneal nerves stem from the ophthalmic lobe of the trigeminal ganglion and thick stromal nerves traverse the anterior limiting lamina to enter the epithelium. These epithelial nerves form a network, the subbasal nerve plexus, of thin, unmyelinated nerve fibers that run parallel to one another [25]. The density of the epithelial nerves increases towards the center of the cornea and they have been shown to respond to chemical, mechanical, and thermal stimulation [26]. Upon epithelial debridement, the thin subbasal nerves are destroyed and regeneration begins toward the wound center. Platelets, neutrophils, and γδ T cells have been shown to aid in this process with the release of specific growth factors and cytokines [27].

Both in animal models and in human studies, vitamin D supplementation has been found to produce therapeutic effects on inflammatory conditions. Therefore, in the current study, a mouse model of corneal epithelial debridement was employed to examine the effect of topical vitamin D treatment on wound closure, neutrophil infiltration, and early nerve regeneration following wounding. The ultimate goal of this work is to provide an increased understanding of vitamin D at the ocular surface and examine its therapeutic potential in this tissue.

## Materials and Methods

### Mouse Model of Corneal Wound Healing

A total of 102 female C57BL/6 mice (8–12 weeks old, Jackson Laboratories, Bar Harbour, ME) were used in these studies. As there is a difference in the rate of wound closure based on sex, only female mice were used in these studies [28]. Prior to wounding, mice were weighed and anesthetized with an intraperitoneal injection of ketamine/xylazine (75mg/7.5mg/Kg body weight) (Vedco, Inc., St. Joseph, MO). Circular epithelial wounds (~2mm in diameter) were made in the center of the right eye with an Algerbrush II (Alger Equipment Co., Lago Vista, TX) under a dissecting microscope. This method has been used previously to debride the corneal epithelium without disruption of the epithelial basement membrane [10,11,17]. All wounding experiments were started at the same time of day to minimize diurnal variations in inflammatory events.

Immediately following wounding and imaging, topical vitamin D (1,25D_3_; Sigma-Aldrich, St. Louis, MO) (10^−7^–10^-9^M) or vehicle (0.02% ethanol/PBS) was applied drop wise (5μl) to wounded corneas. Drops were repeated 5 minutes after the initial application and every 6 hours over the course of the experiments. Eyes were manually held open to avoid blinking for 30 seconds following drop administration. All mice were euthanized by carbon dioxide asphyxiation followed by cervical dislocation at the end of the experiments. This protocol was approved by the Institutional Animal Care and Use Committee at the University of Houston, and adhered to the standards of the Association for Research in Vision and Ophthalmology Statement for the use of animals in ophthalmic and visual research.

### Corneal Wound Imaging

To determine wound size and monitor re-epithelialization, wounded corneas were imaged at the time of wounding and every 6 hours afterwards for 24 hours. Isoflurane anesthesia was administered to each animal before imaging, except at the time of initial imaging. Briefly, mice were placed in an induction box and isoflurane was administered via vaporizer (EZ‐Anesthesia, model EZ‐SA800) at a dosage of 1–4% inhalant and O_2_ flow rate of 0.5–1 liter/minute until there was lack of a reflex to a rear toe pinch. For imaging, 1μl of 1% sodium fluorescein (Sigma-Aldrich) was pipetted onto the central cornea and images were captured with an Olympus stereomicroscope (Model SZX16). For determining percentage of wound closure, wound areas were demarcated and measured using Image J software. The size of the epithelial defect was expressed as a percentage of the original wound area. Results were analyzed with a two-way repeated measure ANOVA and Bonferroni’s test for multiple comparisons with significance set at p<0.05.

### Corneal Whole Mount Imaging

At 18 hours post-wounding, a time demonstrated to correlate to peak neutrophil infiltration [10], mice were euthanized for corneal whole mount processing. Whole eyes were removed and placed in 2% paraformaldehyde for 15 minutes. Corneas were then dissected and fixed for an additional 45 minutes. Following blocking and permeabilization (2% BSA/0.01% Triton-X100 in PBS), corneas were incubated overnight at 4°C with 10μg/ml fluorophore-conjugated antibodies: FITC-conjugated anti-Ly6G (to detect neutrophils) (clone 1A8, BD Pharmingen, San Diego, CA), NorthernLights™ NL557-conjugated anti-β-III tubulin (to detect nerves) (R&D Systems, Minneapolis, MN), and DAPI (4',6-diamidino-2-phenylindole, Sigma-Aldrich) to visualize nuclei. Corneas were radially cut to flatten, mounted onto slides with Airvol (Celanese, Dallas, TX, courtesy of Dr. A. Burns) and imaged on a DeltaVision Core microscope (Applied Precision, Issaquah, WA).

**Fig 1:**
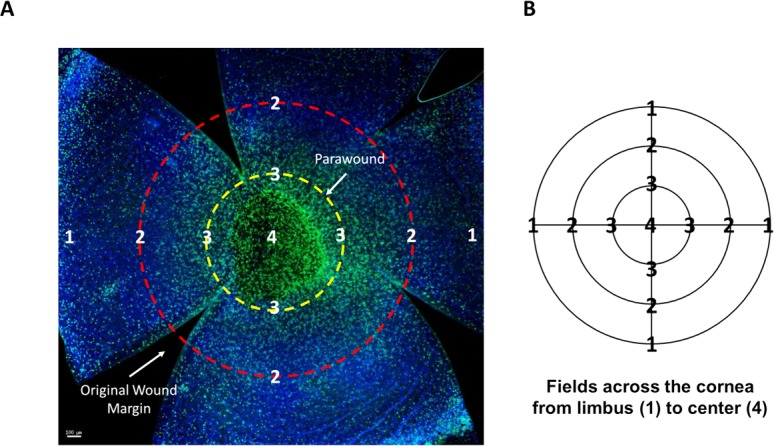
Corneal whole mount imaging. (A) Example of a whole mount, 18 hours post-wound, stained with Ly6G (green) to visualize infiltrating neutrophils and DAPI (blue). Radial cuts divide the cornea into four petals, enabling the cornea to flatten under the coverslip. The wound area is visible in the central cornea surrounded by the parawound region (yellow). The location of the original wound margin is demarcated (red). Scale bar = 100μm (B) Each cornea was measured from the limbus across the center to the opposite limbal region using x- and y- image coordinates and images were taken at even intervals, designated by points 1 through 4.

Full thickness images were captured from seven fields of view across the diameter of the cornea, centered at evenly spaced intervals, from limbus to limbus on the opposite side (Fig 1). x- and y- image coordinates were used to calculate the distance between points. Each image was deconvolved five iterations to improve resolution and reduce blur using SoftWorx software (Applied Precision) [29]. For neutrophil infiltration, nuclei were counted within a square morphometric frame (150 μm in length) (S1 Fig). Both DAPI and FITC-Ly6G staining were used to identify neutrophils, with distinctive “donut-shaped” nuclei (polymorphonuclear), which were located predominantly in the anterior stroma, as previously reported [13] and only those cells which fell within the frame or on the accepted line were counted. A similar method was used to count dividing basal epithelial cells, which were identified by DAPI nuclear staining, enabling visualization of condensed chromosomes that occur during mitosis. For relative nerve-density determination, a 10x10 grid was overlaid on each image and the presence or absence of stained subbasal epithelial nerves in each square of the grid was recorded to obtain a percentage of squares containing nerves stained with neuron-specific anti-β-III tubulin (S2 Fig).

### Corneal RNA Collection and RT-PCR

Whole corneas were collected for RT-PCR analyses of gene expression 12 and 24 hours post-wounding. Following euthanasia, eyes were removed and corneas harvested with the aid of a dissecting microscope. Eight to ten corneas (from 4–5 mice) were pooled from the same treatment group for each sample. Samples were homogenized on ice and RNA was extracted with an Ambion ToTALLY RNA Total RNA Isolation kit (Life Technologies, Grand Island, NY), using two sequential phenol:chloroform extractions for purification. RNA concentration was quantified with a NanoDrop 2000 spectrophotometer (Thermo Scientific, Wilmington, DE) and reverse transcribed using an AffinityScript cDNA synthesis kit (Agilent Technologies, Santa Clara, CA). Real-time PCR was performed using intron-spanning primers (Table 1) and Brilliant II SYBR Green QPCR master mix (Agilent Technologies). All samples were normalized to GAPDH and control samples served as the calibrator for relative quantity determination using the ΔΔCT method.

**Table 1:** Primer sequences for real-time PCR.

Gene name	Forward Primer	Reverse Primer	NCBI Reference Sequence
*CXCL1*	5’- TGCACCCAAACCGAAGTC-3’	5’- GTCAGAAGCCAGCGTTCACC-3’	NM_008176.3
*CXCL2*	5’-TGTCAATGCCTGAAGACCCTGCC-3’	5’-AACTTTTTGACCGCCCTTGAGAGTGG-3’	NM_009140.2
*IL-1α*	5’- CAGGGCAGAGAGGGAGTCAAC-3’	5’- CAGGAACTTTGGCCATCTTGAT-3’	NM_010554
*Il-1β*	5’- GCAACTGTTCCTGAACTCAACT-3’	5’- ATCTTTTGGGGTCCGTCAACT-3’	NM_008361
*VEGFA*	5’- GTCCTGTGTGCCGCTGATG-3’	5’- GCTGGCTTTGGTGAGGTTTG-3’	NM_001025250
*TGFβ1*	5’-CTTCAATACGTCAGACATTCGGG-3	5’- GTAACGCCAGGAATTGTTGCTA-3’	NM_011577
*TGFβ2*	5’- CTTCGACGTGACAGACGCT-3’	5’-TTCGCTTTTATTCGGGATGATG-3’	NM_009367
*TNFα*	5’- ACTGAACTTCGGGGTGATCG-3’	5’- TGATCTGAGTGTGAGGGTCTGG-3’	NM_013693.3
*PDGFα*	5’- TGGCTCGAAGTCAGATCCACA-3’	5’- TTCTCGGGCACATGGTTAATG-3’	NM_008808

### ELISA

Levels of CXCL1, CXCL2, TNFα, IL1β, and VEGF were determined 12 and 24 hours after wounding. Whole corneas were harvested and protein isolated as previously described [30]. Eight corneas (from 4 mice) per group were pooled and homogenized in 1ml PBS with protease inhibitors (cOmplete, mini tablets, Roche Diagnostics, Indianapolis, IN). Cells were then lysed through three freeze-thaw cycles and centrifuged at 10,000g. Total protein concentrations were determined in supernatants by BCA protein assay (Life Technologies) and target protein expression quantified in duplicate using DuoSet ELISA kits (R&D Systems). Results were expressed as amount of target protein per mg total protein from each sample.

### Human Organ Culture Model

Human cornea organ culture wounding was performed as previously described [31]. Briefly, cadaveric corneas, supplied by Saving Sight (St. Louis, MO), were received within five days of donor death, stored in corneal preservation media (Life 4°C, Numedis, Inc., Isanti, MN), and shipped overnight at 4°C. Upon receipt, corneas with epithelial defects were excluded and only corneas with an intact epithelium were used in these experiments. Corneas were placed epithelial side up in a culture dish supported by a 0.5% agar/M199 mold. In order to examine how gene expression was affected by wounding, the epithelium was removed and allowed to regrow for 48 hours. Briefly, corneas were gently marked with a 6mm trephine and the epithelium was removed from the corneas by scraping (initial sample), allowed to regrow, and collected again after 48 hours (regrown sample). RNA was isolated and analyzed by RT-PCR for expression of CYP27B1, the hydroxylase that converts vitamin D to its active form, 1,25D_3_. For wound closure studies, 1,25D_3_ (10^-7^M) (right eye, OD) or vehicle (0.02% ethanol/PBS) (left eye, OS) was applied drop wise to wounded corneas every 6 hours for 24 hours and the wound area determined by fluorescein staining, as in the mouse wounding model (described above). Results were analyzed with a two-way repeated measure ANOVA and Bonferroni’s test for multiple comparisons with significance at p<0.05. Five corneas for each treatment, vehicle or 1,25D_3_, were used in the analysis.

## Results

### Topical vitamin D slows corneal wound closure in the initial 18 hours

**Fig 2:**
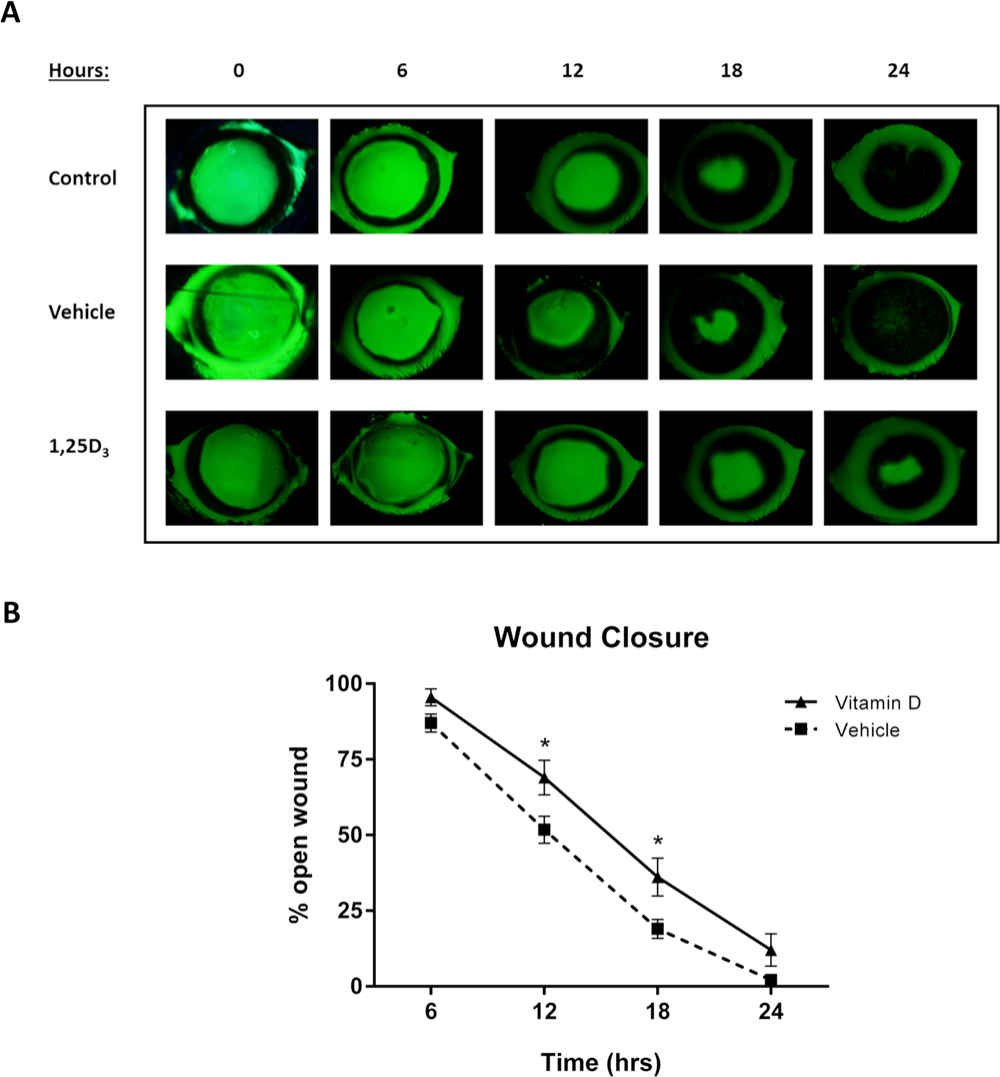
*In vivo* wound closure is delayed with vitamin D treatment. Mice were wounded and treated with vehicle (0.02% ethanol/PBS) or 1,25D_3_ (10^-7^M) every 6 hours or left untreated (control) for 24 hours. (A) Corneal wound areas were monitored by fluorescein staining every 6 hours. (B) Wound area remaining open was determined as a percentage of original wound area. Data represent mean ± SEM and were analyzed with two-way repeated measures ANOVA and Bonferroni’s correction for multiple comparisons. * = p<0.05 (comparison between vehicle and vitamin D treatments; n = 9 mice/group).

To determine the effect of vitamin D on wound closure, topical 1,25D_3_ (10^-7^M, S3 Fig) was administered every 6 hours to wounded corneas and re-epithelialization was evaluated over 24 hours. 1,25D_3_ treated mice had a significantly delayed rate of wound closure, indicated by a greater percentage of wound remaining open, at both 12 and 18 hours compared to vehicle treatment (p<0.05) (Fig 2). Following epithelial debridement, wounds from normal mice were nearly completely re-epithelialized within 24 hours [10], as was seen with vehicle treatment, having only 2.16% of the wound area remaining open. 1,25D_3_ treated wounds were not completely closed at this time (12.01% open), however this difference did not reach statistical significance. Vehicle treatment alone did not alter the rate of wound closure compared to untreated control wounds.

### Vitamin D treatment does not affect basal epithelial cell division

**Fig 3:**
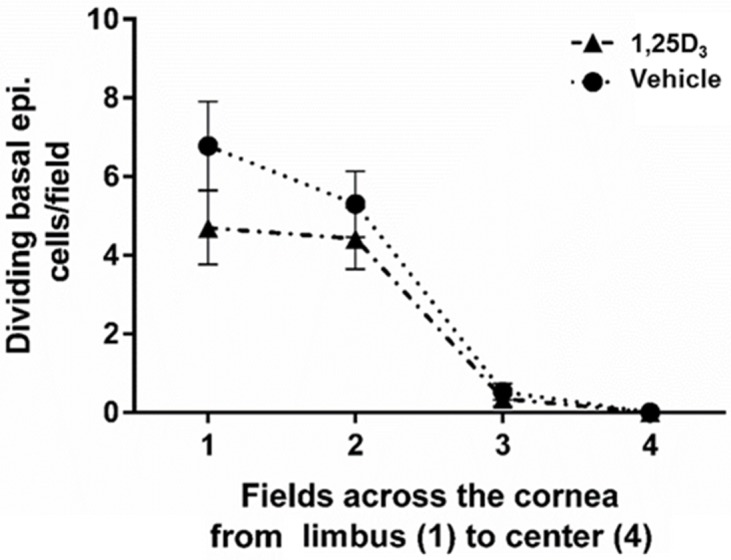
Basal epithelial cell division across the wounded cornea at 18 hours after wounding. Nuclei of basal epithelial cells were identified with DAPI staining. The graph represents the average number of mitotic nuclei counted per field in vehicle or 1,25D_3_ treated corneas 18 hours after wounding. (n = 5 corneas/group).

Re-epithelialization of a corneal wound involves both migration and division of basal epithelial cells to cover the wound. Because vitamin D delayed wound closure, the amount of basal epithelial cell division was quantitated to determine if this process was affected by treatment. At 18 hours after epithelial debridement, there was no significant change in dividing epithelial cell numbers between vehicle and 1,25D_3_ treatment groups across the cornea (Fig 3). This suggests that the delay seen in corneal wound healing with vitamin D was not due to reduced basal cell division. However, the power of this experiment was only 0.60, indicating that a difference might arise with an increased sample size.

### Neutrophil infiltration into the wound area is increased by vitamin D treatment

**Fig 4:**
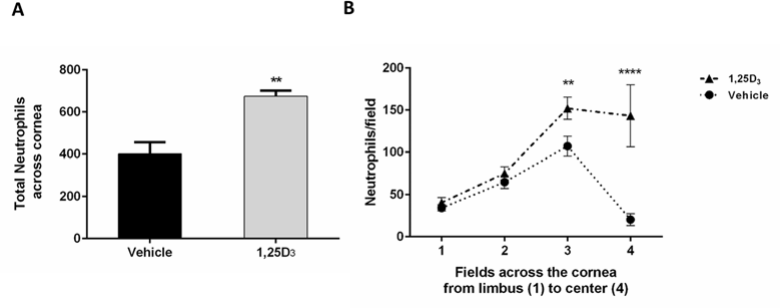
Vitamin D treatment increases neutrophil infiltration in wounded corneas. (A) Total neutrophil counts across the cornea at 18 hours post-wound from fields 1 through 4. Data represent mean ± SEM and were analyzed with Student’s two-tailed t-test, p<0.01 (n = 6). (B) Neutrophil counts per field in vehicle or vitamin D-treated corneas at 18 hours after wounding. Statistical analysis was by two way ANOVA with Bonferroni’s test for multiple comparisons. p<**0.01, ****<0.0001 (n = 6).

Neutrophil extravasation and emigration into the cornea is an important part of the wound healing process and neutrophil depletion causes a delay in wound healing [10]. Therefore, we next examined neutrophil infiltration in wounded corneas at 18 hours post-wounding, the time of peak neutrophil accumulation in corneas under control conditions [10]. Vitamin D treatment increased the relative number of total neutrophils in the wounded corneas by 40% compared to vehicle (674 vs. 401; p<0.01) (Fig 4A). Specifically, there were more neutrophils in the parawound (p<0.01) and wound center (p<0.0001) regions with 1,25D_3_ treatment (Fig 4B).

### Levels of CXCL1 are increased at 12 hours after wounding with vitamin D treatment

**Fig 5:**
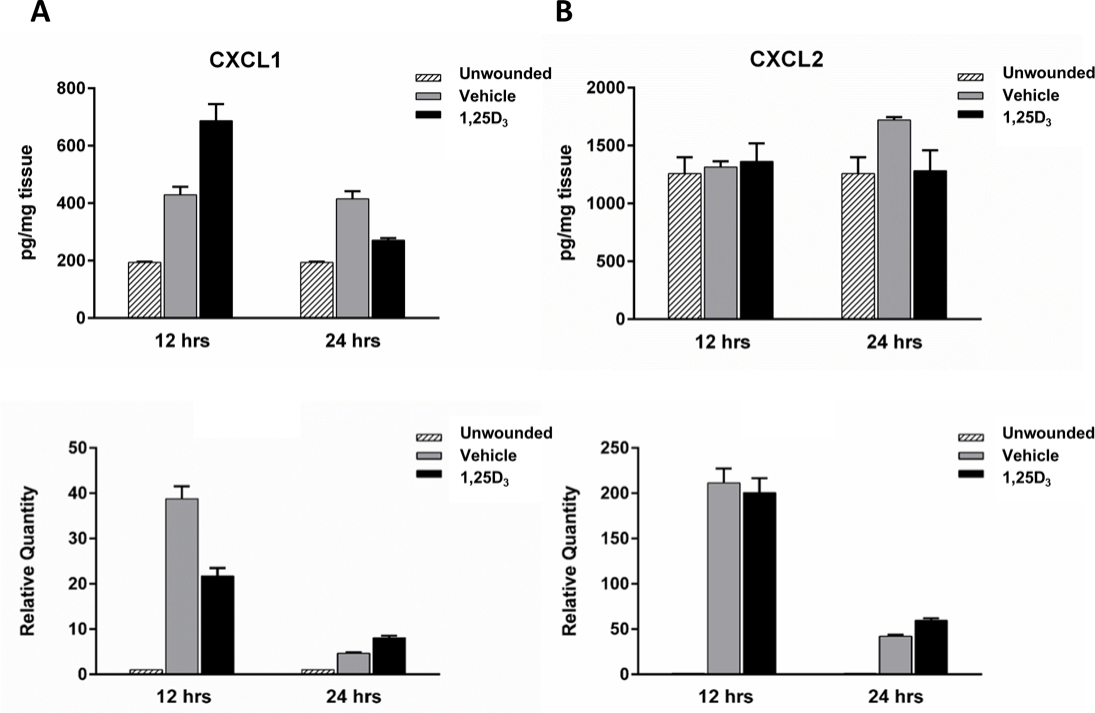
Chemokine CXCL1 and CXCL2 expression in wounded corneas. CXCL1 (A) and CXCL2 (B) expression was determined in corneal homogenates following 12 and 24 hours of wounding. For protein analysis (top row), 8 corneas were isolated per time point per treatment group for each experiment, were pooled, and total corneal protein was collected for ELISA analysis of chemokine expression. Graphs are representative data from one experiment (n = 2, 24hrs; n = 1, 12hrs) showing the mean and ± SD of duplicate technical repeats. For RNA analysis (bottom row), relative gene expression was determined by RT-PCR analysis. 10 individual corneas were isolated per time point per treatment group for each experiment. Graphs are representative data from one experiment (n = 3, 24hrs; n = 1, 12hrs) showing the mean ± SEM of triplicate technical repeats.

Neutrophils migrate into the cornea from the limbal vessels in response to chemotactic signals resulting from injury. As vitamin D treatment increased neutrophil migration into the wounded area 18 hours after epithelial debridement we examined the expression of CXCL1 and CXCL2, molecules known to be expressed in the cornea and to enhance neutrophil chemotaxis [32,33], in wounded corneas. At 12 hours after wounding, prior to the 18 hour peak of neutrophils in the cornea, there was a 38% increase in CXCL1 protein expression with vitamin D treatment, above the increase seen with vehicle (Fig 5A top). However, there was no change at this time point with CXCL2 (Fig 5B top). By 24 hours post-wounding, after the peak neutrophil infiltration, there was a decrease in CXCL1 expression with vitamin D, below the level of vehicle treatment. In addition, while CXCL2 levels were increased at 24 hours with vehicle treatment, compared to unwounded controls, there was no increase in CXCL2 expression in the vitamin D treated corneas. Examining gene expression, there was no change in CXCL2 relative RNA quantity with treatment at either time point (Fig 5B bottom) but a decrease in CXCL1 at 12 hours (Fig 5A bottom). Wounded corneas, regardless of treatment, had increases in both CXCL1 and CXCL2 relative to unwounded corneas at both time points (bottom).

### Vitamin D does not affect early nerve regeneration or VEGF expression in the wounded corneas

Upon epithelial debridement, the thin subbasal nerves are destroyed in the wounded area. These nerves regenerate during wound healing, however, they only reach 65% of their original density by 28 days after wounding [24]. Vascular endothelial growth factor (VEGF) has been shown to be important in promoting nerve regeneration following corneal epithelial debridement and is upregulated after wounding [24,34]. In other tissues, vitamin D has been shown to modulate VEGF levels, with differing responses dependent on cell type and tissue condition [35–37]. Therefore, we next examined if there was a change in relative nerve densities and VEGF expression between treatment groups early in the regeneration process.

**Fig 6:**
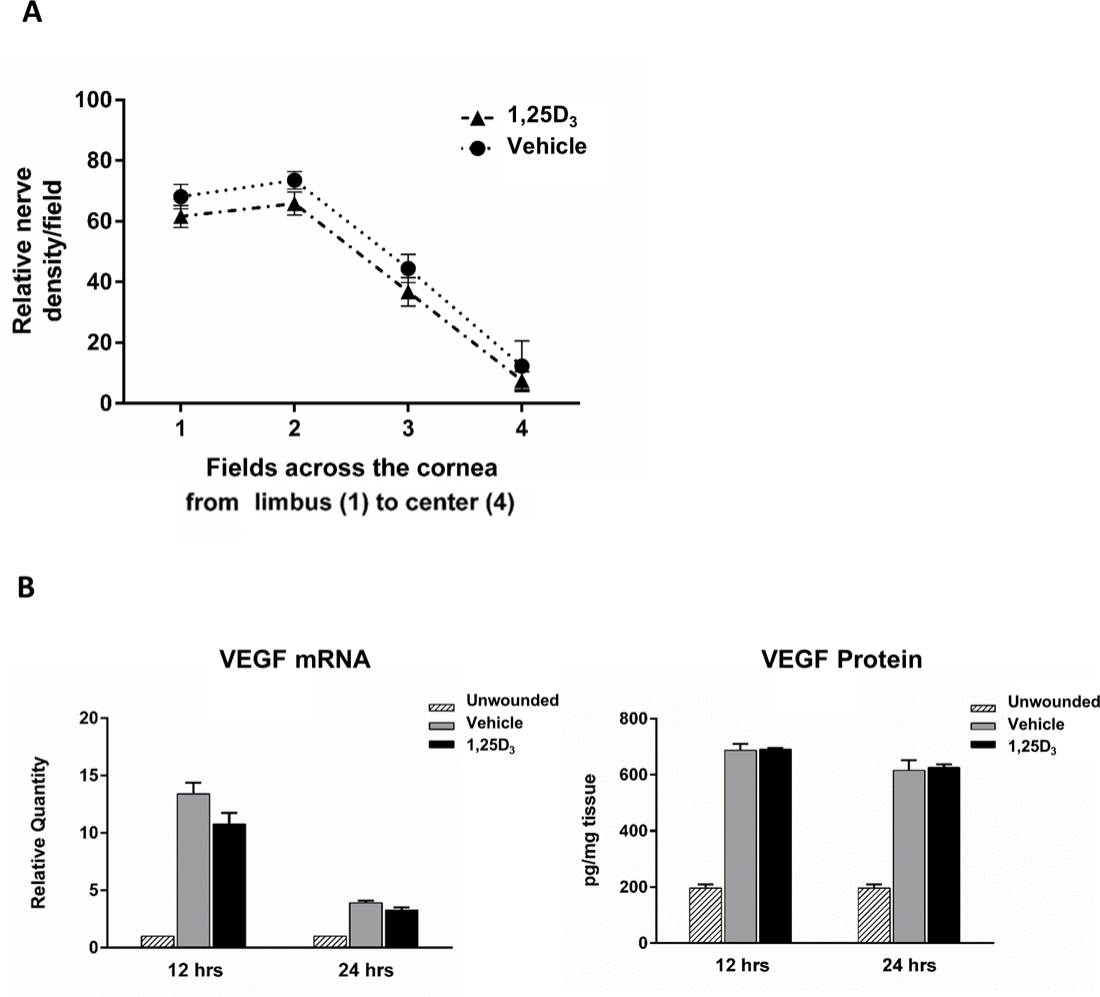
Topical vitamin D treatment does not change subbasal nerve density or VEGF expression following wounding. (A) Corneal whole mounts were stained with anti-tubulin β III and subbasal nerves counted in a 10x10 morphometric grid to determine relative nerve densities. The graph represents the average nerve density per field in vehicle or 1,25D_3_ treated corneas 18 hours after wounding. (n = 5 corneas/group). (B) VEGF expression was determined in corneal homogenates 12 and 24 hours after wounding. For RNA analysis (left), relative expression was determined by RT-PCR analysis. Samples represent 10 pooled corneas per group at each time point. Graphs are representative data from one experiment (n = 3, 24hrs; n = 1, 12hrs) showing the mean ± SEM of triplicate values. For protein analysis (right), total corneal protein was collected and ELISA performed. Data represent 8 pooled corneas per group at each time point. Graphs are representative data from one experiment (n = 2, 24hrs; n = 1, 12hrs) showing the mean of duplicate values.

There was no significant difference in subbasal nerve density with 1,25D_3_ treatment compared to vehicle in any corneal region (Fig 6A). In addition, there was no change in either VEGF mRNA or protein expression 12 and 24 hours after wounding with topical 1,25D_3_ treatment_._ There was however, the expected increase in VEGF when compared to unwounded controls (3-fold increase) (Fig 6B). These data suggest that vitamin D, while delaying wound closure, does not slow the initial nerve regeneration after epithelial debridement or affect VEGF levels.

### Vitamin D treatment does not alter expression of cytokines after wounding

**Fig 7:**
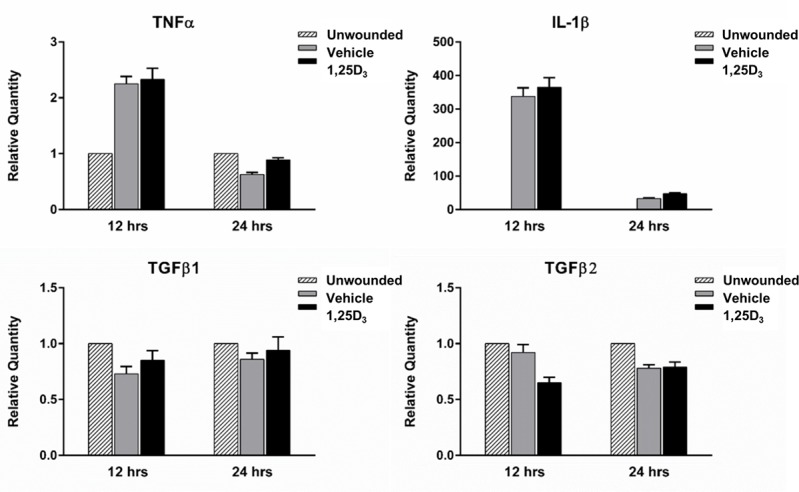
Cytokine expression at 12 and 24 hours after corneal wounding. TNFα, IL-1β, TGFβ1, and TGFβ2 expression was determined in corneal homogenates following 12 and 24 hours of wounding by RT-PCR. Samples represent 10 pooled corneas per group at each time point. Graphs are representative data from one experiment (n = 3, 24hrs; n = 1, 12hrs) showing the mean ± SEM of triplicate values.

Cytokines play an important role in integrating various aspects of the wound healing process. Inflammatory mediators, at controlled levels, aid in advancing re-epithelization and serve as communication bridges, relaying signals between immune cells, epithelial cells, and stromal keratocytes. Vitamin D is able to modulate cytokine expression during inflammation in a number of tissues, including the cornea [38,39]. Therefore, cytokine expression was compared between treatment groups after wounding. At both 12 and 24 hours post-wound, topical 1,25D_3_ did not change corneal mRNA expression of TNFα, IL-1β, TGFβ1, or TGFβ2 compared to vehicle (Fig 7). 1,25D_3_ also did not influence TNFα protein expression at 12 hours, however at 24 hours, there was a decrease in TNFα with 1,25D_3_ (S4 Fig).

### Antimicrobial peptide expression is induced by vitamin D treatment 12 hours after wounding

**Fig 8:**
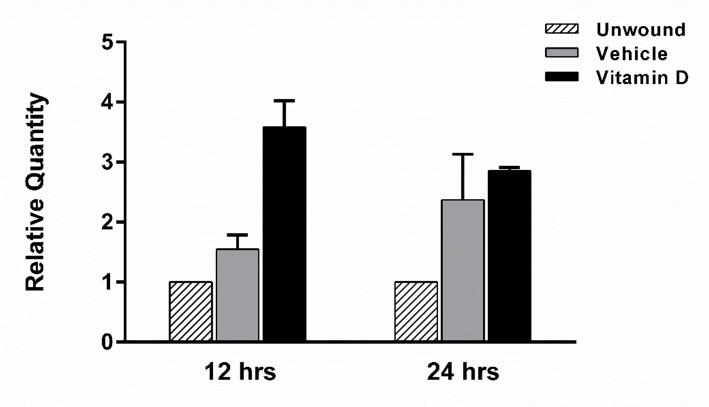
Vitamin D increases the expression of CRAMP in wounded corneas. CRAMP expression was determined in corneal homogenates following 12 and 24 hours of wounding by RT PCR. 10 individual corneas were isolated and pooled per time point per treatment group for each experiment. Graphs are representative data from one experiment (n = 2, 24hrs; n = 1, 12hrs) showing the mean ± SEM of triplicate technical repeats.

Similar to cytokines, the antimicrobial peptide CRAMP has been shown to be important in the cornea during inflammation [40]. The human homologue to CRAMP, LL-37, is important for defense of the cornea against infection and increases migration of human corneal epithelial cells [31]. At 12 hours after epithelial debridement, 1,25D_3_ treated corneas expressed an increased level of CRAMP (3.58-fold) compared to both unwounded and vehicle treated animals (Fig 8). This increase could potentially offer protection to the exposed cornea while the wound is re-epithelializing.

### Vitamin D does not change the rate of wound closure in a human organ culture model but does increase CYP27B1 expression

**Fig 9:**
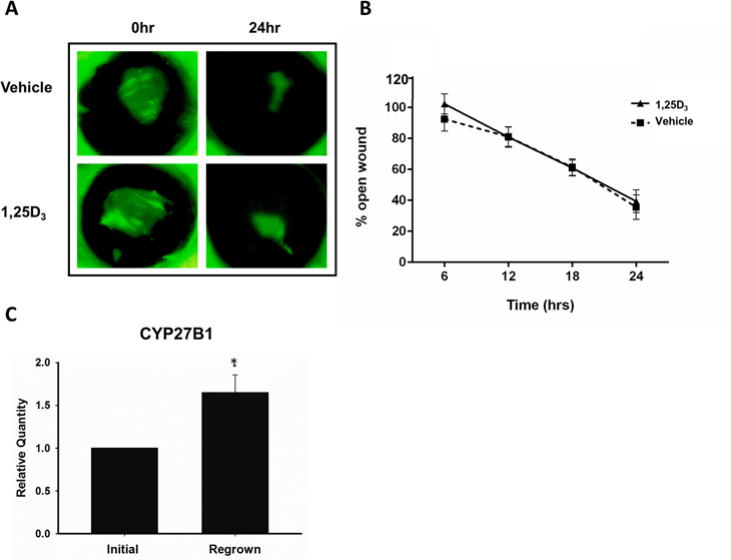
Topical vitamin D treatment does not affect wound closure rate in a human organ culture wounding model but wounded corneas express a higher level of the vitamin D activating enzyme, CYP27B1. **(**A, B) Corneal wounds were monitored by fluorescein staining every 6 hours for 24 hours. Data were analyzed with repeated measures ANOVA and Bonferroni’s correction for multiple comparisons. (n = 5) (C) Corneal epithelium was collected at the time of wounding (initial) and after 48 hours of regrowth (regrown) and CYP27B1 expression was determined by RT-PCR. Data were analyzed by Student’s two-tailed t-test. * = p<0.05 (n = 4).

In addition to a mouse model of wounding, we also wanted to examine the effects of vitamin D treatment in human corneal wound healing. Donor corneas were wounded by epithelial debridement and wound closure was followed over 24 hours. In this *ex vivo* model, vitamin D treatment did not influence the rate of wound closure (Fig 9A and 9B). There was no statistical difference in percent open wound at any time point between 1,25D_3_ and vehicle treatment. There was however, a significant increase in CYP27B1 expression, a hydroxylase which converts 25D_3_ to the biologically active 1,25D_3_, after 48 hours of wound healing in the human corneas (1.65-fold increase; p<0.05) compared to pre-wounded epithelium. (Fig 9C).

## Discussion

The cornea is the transparent anterior covering of the eye that refracts entering light for vision and serves to protect the underlying ocular tissues from damage. After injury, it is essential to have a well-coordinated corneal wound healing response to prevent loss of transparency and hence vision and to maintain the protective barrier. Corneal wound healing is a complex process that involves epithelial cell division and migration, death of stromal keratocytes, nerve regeneration, and a localized inflammatory response resulting in infiltration of immune cells and platelet extravasation from limbal vessels. All of these components are necessary for efficient corneal wound healing. As vitamin D is well recognized to modulate inflammatory events in many tissues, we sought to determine if vitamin D treatment influences the corneal wound healing response. We observed that mice treated with topical vitamin D had a delay in sterile wound closure, possibly the result of an acute increase in neutrophil infiltration.

Following debridement of a 2 mm diameter circular area, re-epithelialization of the murine cornea takes approximately 24 hours, resulting in complete covering of the wounded area with basal epithelial cells [11,41]. Re-epithelialization involves first the migration of epithelial cells to cover the wound and then a proliferative phase to aid in re-stratification. Immediately after wounding, cell division is inhibited in the wound area, to allow for efficient migration [18,42,43]. Our data is in agreement with this model of corneal-wound healing, with almost no basal epithelial cell proliferation in the central cornea, within the wounded area at 18 hours post-wound, a time when the wound is still being re-epithelialized, in either vitamin D or vehicle treated mice. There was also no significant difference in basal epithelial cell division in the peripheral corneal regions between the treatment groups. However, as noted (Fig 3), a reduction in cell proliferation with vitamin D treatment might become apparent with an increased sample size, as there appeared to be a slight decrease in proliferation with vitamin D. If confirmed, this could account for part of the delay in wound closure, seen with vitamin D treatment.

There was a significant delay in wound closure at both 12 and 18 hours with topical vitamin D treatment, and wounds still remained open at 24 hours, although not statistically significantly more so than in controls. This delay could also potentially be a result of decreased epithelial cell migration. Notably, a recent study demonstrated similar results when 1,25D_3_ was applied to mouse corneas during an *ex vivo* alkali burn model, with delayed re-epithelialization resulting from vitamin D treatment.[44] Interestingly, vitamin D receptor (VDR) knockout mice also had delayed wound healing in a corneal abrasion model compared to wild type controls.[45] In this study, normal wound healing rates were restored in the knockout animals with a high-lactose, Ca^2+^, and PO_4_^−^ diet, indicating that vitamin D’s calcemic effects were important for corneal wound healing. It would therefore be relevant to examine how vitamin D influences calcium signaling events during re-epithelialization in further studies.

In the human organ culture model of wounding, vitamin D did not change the wound closure rate, despite the finding that *in vitro*, vitamin D treatment decreased human corneal epithelial cell proliferation (S5 Fig). Importantly, however, this *ex vivo* model does not include contributions from the ocular surface system, including the tear film, lacrimal glands, eyelids, and conjunctival tissues, which all work together *in vivo* during ocular surface and corneal damage. In particular, the human organ culture model does not account for the influence of infiltrating inflammatory and immune cells, which are critical for effective wound healing.

Epithelial debridement results in an acute inflammatory response that is necessary for efficient wound healing and re-epithelialization. Animal models of corneal injury have demonstrated that wounding triggers the infiltration of immune cells into the cornea from the limbal vessels. This infiltration is necessary for proper re-epithelialization, as defects in neutrophil trafficking result in delayed wound closure [10–12,17]. In addition to a loss of neutrophil infiltration, excess accumulation also results in delayed re-epithelialization [13,18]. This demonstrates that while a limited inflammatory infiltration is beneficial, too many neutrophils impair corneal healing. We therefore examined neutrophil infiltration into the corneal stroma in our model. With topical vitamin D treatment, in addition to delayed wound closure, there was an increase in neutrophil accumulation throughout the cornea and specifically in the wounded area at 18 hours. The increase in neutrophils seen with vitamin D could in part be a result of an increase in chemotactic signals. CXCL1 (C-X-C motif ligand 1, GRO1, KC) a chemokine that recruits neutrophils to sites of injury and inflammation [32,33] and has been shown to be elevated following corneal abrasion [10]. There was a greater amount of CXCL1 protein in wounded corneas with vitamin D treatment, just prior to the peak in neutrophils, potentially contributing to the greater numbers of neutrophils in the corneas of these mice at this time. There is an apparent discrepancy between the CXCL1 RNA and protein data; while protein expression is higher with vitamin D treatment at 12 hours post-wound, CXCL1 gene expression is decreased at this time relative to vehicle control (Fig 5). Vitamin D could be upregulating CXCL1 mRNA rapidly (prior to 12 hours), resulting in an increase in CXCL1 protein translation and secretion (evident at 12 hours), then decreases CXCL1 gene regulation with time. It would therefore be important to examine earlier time points, immediately after wounding, to determine vitamin D’s effect on early chemokine gene expression. It would also be interesting to further explore the mechanism behind the decrease in CXCL1 protein at 24 hours with vitamin D treatment and examine if this is a result of increased degradation, decreased secretion, or a decrease in protein translation, and to examine this effect on neutrophil infiltration at later time points.

Previous studies have shown that an increase in neutrophil infiltration disrupts wound healing and re-epithelialization [13], however the exact mechanism of this remains uncertain, as does the amount of neutrophil infiltration that normally occurs in a human corneal epithelial wound. Excess neutrophil accumulation in the wounded area could potentially block epithelial cell migration into the area and therefore result in a delay in wound closure. However, the increase in neutrophil infiltration with vitamin D would be beneficial in a non-sterile wound environment, where neutrophils would be an important mediator of infection clearance. One explanation for the increase in neutrophils with topical vitamin D, which was not examined in this study, is disruption of neutrophil apoptosis. Neutrophils undergo rapid programmed cell death, or apoptosis, following infiltration to the site of inflammation [46]. If this process is inhibited, there would be a greater number of neutrophils remaining in the wounded area. Another interesting situation to further explore would be the effect of vitamin D on other inflammatory cells that are known to migrate into the wounded area. Local dendritic cells, Langerhan’s cells, are needed for re-epithelization, migrating with epithelial cells towards the wound center [19]. NK cells and γδ T lymphocytes also infiltrate into the cornea and influence recovery. γδ T cell-deficient mice have delayed wound re-epithelialization and a decrease in corneal neutrophils and limbal platelet accumulation [12]. Depleting NK cells in an epithelial abrasion model increases neutrophil infiltration while negatively affecting re-epithelialization and nerve regeneration. In this study, Liu et al. (2012) suggest that NK cells act to limit acute inflammatory events. *In vitro* studies suggest that vitamin D has a negative effect on NK cell development [47]. Therefore, investigation into vitamin D’s influence of NK cells in wounding is warranted.

In order to further dissect the effect of vitamin D treatment on the wound healing process, nerve regeneration and cytokine expression were also examined in the wounded corneas. Nerves release trophic factors, which help to maintain the health of the corneal epithelium and stroma. Various factors play a role in nerve regeneration, including VEGF, produced by the wounded epithelium and infiltrating neutrophils [27]. However, there was no difference in either nerve density in the wounded corneas or VEGF expression with topical vitamin D treatment. Cytokines also play an integral role in the localized inflammatory reaction following wounding [43,48,49]. Disruption in TGFβ signaling delays corneal re-epithelialization [43,50,51]. IL-1 and TNFα also are important mediators of corneal injury induced inflammation, influencing both proliferation and migration of epithelial cells [48,49]. Mice deficient in these cytokines have compromised corneal wound healing, with decreased dendritic cell migration in the wounded epithelium [19,52,53]. IL-1 is released from the injured epithelium and also induces keratocyte cell death, a coordinated aspect of the wounding process [14,15]. Interestingly, when NF-κβ signaling is disrupted, corneal wound healing is delayed, causing a decrease in pro-inflammatory cytokines and a reduction in cell migration [54]. However, levels of IL-1β, TNFα, TGFβ1, and TGFβ2 were not changed with vitamin D treatment in these experiments.

While this study did not detect changes in VEGF/cytokines with vitamin D treatment, it is important to note that whole corneas were used in the analysis. Therefore, the results reflect general corneal expression, with contributions from epithelial, stromal, and immune cells. Subtle differences, for example, in epithelial expression of cytokines or neutrophil expression of VEGF, caused by vitamin D, might have been masked. This especially merits consideration, given the increase in neutrophils with vitamin D treatment. It would therefore be interesting to examine cytokine expression in the isolated epithelium and compare this to results from whole corneal expression.

Another aspect of wound healing is the protection of injured cornea from infection during re-epithelialization. Antimicrobial peptides (AMP) are small cationic peptides which have been shown to be expressed during wound healing, providing a line of defense against pathogens [40,55–59]. One of these AMPs, cathelicidin (murine CRAMP/ human LL-37), was first identified in neutrophils, however, now it is known to be expressed in the corneal epithelium [60]. In addition to its broad range killing ability, LL-37 is also able to modulate inflammatory signals, bind LPS, and potentiate the wound healing process. It has also been shown to be chemotactic for neutrophils and T lymphocytes [61] and increases the migration of human corneal epithelial cells in culture [31]. Vitamin D augments the production of LL-37 in various human tissues; however it is unclear if CRAMP, the murine orthologue, is influenced by vitamin D. In our study, at 12 hours after the initial wound, vitamin D increased CRAMP RNA expression. Therefore, while delaying early wound closure, vitamin D treatment could simultaneously be enhancing protection of the exposed epithelium, preventing infection of the underlying tissue. Both the increase in CRAMP and neutrophils with vitamin D could be of benefit in an infected wound.

Vitamin D is known to modulate inflammation in a variety of tissues and is protective against the development several ocular diseases [62]. The current study highlights the fact that various factors influence the effects of vitamin D during inflammation and that its role in inflammation is complex. The response to inflammation can change based on the cell types involved, the source of immune stimulus, the tissue microenvironment, timing of vitamin D administration, vitamin D concentration, and the relative expression of activating and inactivating hydroxylases. The effect of topical vitamin D on corneal wound healing is an important area of further research and should be considered when evaluating vitamin D as a therapeutic option during inflammatory conditions.

## Supporting Information

S1 Fig Neutrophil counts. Morphometric counting frame with the accepted line in green and forbidden line in red dashes. Neutrophil “donut-shaped” nuclei were counted using DAPI (blue) and cross-checked with FITC-labeled Ly6G staining (green) throughout the z-stack in each image. Scale bar = 20μm.
(TIF)

S2 Fig Nerve relative density. The left panel shows the 10x10 grid used to count subbasal epithelial nerves stained with NorthernLights™ NL557-conjugated anti-β-III tubulin (red). In this image, 57 out of 100 boxes contain a subbasal nerve fiber. A large stromal nerve can be seen beneath the plane of the thin subbasal nerves. On the right, basal epithelial cells are visible, stained with DAPI (blue). Scale bar = 20μm.
(TIF)

S3 Fig Vitamin D increases the percentage of open wound at 18 hours after epithelial debridement. An initial wounding experiment was performed to test the effect of a range of vitamin D concentrations (10^−7^ to 10^-9^M) on corneal wound closure. Mice were imaged immediately after corneal wounding and at 18 hours post-wound with fluorescein staining to visualize wound area. Mice received topical vehicle (0.02% ethanol/PBS) or 1,25_3_ (10^−7^, 10^−8^, or 10^-9^M) twice at the time of wounding and every 6 hours through 18 hours. Wound area remaining open was determined as a percentage of original wound area. Data represent mean ± SEM and were analyzed with one-way ANOVA and Bonferroni’s correction for multiple comparisons, p<*0.05 (n = 3 mice/group).
(TIF)

S4 Fig Vitamin D treatment decreases TNFα protein expression at 24 hours after corneal debridement. To determine vitamin D’s effect on pro-inflammatory cytokine expression following epithelial wounding, TNFα protein levels were determined in corneal homogenates 12 and 24 hours after wounding by ELISA. At 12 hours post-wounding, there was no change in TNFα protein with treatment. However, at 24 hours, vitamin D treatment decreased protein expression in corneal homogenates compared to vehicle (700pg/mg compared to 391pg/mg). Data represent 8 pooled corneas per group at each time point. Graphs are representative data from one experiment (n = 2, 24hrs; n = 1, 12hrs) showing the mean of duplicate values.
(TIF)

S5 Fig Cell viability following vitamin D treatment. Human corneal epithelial cells (hTCEpi [63]) were plated in 96 well plates and treated with 1,25D_3_ (10^-7^M). Following 24 hours incubation, 0.5mg/ml MTT (3-(4, 5-dimethylthiazolyl-2)-2, 5-diphenyltetrazolium bromide) was added to each well and cells were incubated for an additional 2 hours at 37°C. Colorimetric changes were measured at wavelength 590 on a spectrophotometer and OD values normalized to untreated control cells. 0.02% benzalkonium chloride in PBS (BAC) was used as a positive control, indicating loss of cell viability. Data represent mean +/- SEM of 4 independent experiments. Statistical analysis was by Student’s t-test with * p = 0.022.
(TIF)

## References

[1] DeLuca HF. The metabolism and functions of vitamin D. Adv Exp Med Biol. 1986;196: 361–375. 3012979 10.1007/978-1-4684-5101-6_24

[2] Lin R, White JH. The pleiotropic actions of vitamin D. BioEssays News Rev Mol Cell Dev Biol. 2004;26: 21–28. 10.1002/bies.10368 14696037

[3] White JH. Vitamin D signaling, infectious diseases, and regulation of innate immunity. Infect Immun. 2008;76: 3837–3843. 10.1128/IAI.00353-08 18505808 PMC2519414

[4] Hewison M. Vitamin D and the immune system: new perspectives on an old theme. Endocrinol Metab Clin North Am. 2010;39: 365–379, table of contents. 10.1016/j.ecl.2010.02.010 20511058 PMC2879394

[5] Tsoukas CD, Provvedini DM, Manolagas SC. 1,25-dihydroxyvitamin D3: a novel immunoregulatory hormone. Science. 1984;224: 1438–1440. 6427926 10.1126/science.6427926

[6] Cantorna MT, Snyder L, Lin Y-D, Yang L. Vitamin D and 1,25(OH)2D regulation of T cells. Nutrients. 2015;7: 3011–3021. 10.3390/nu7043011 25912039 PMC4425186

[7] Wang T-T, Nestel FP, Bourdeau V, Nagai Y, Wang Q, Liao J, et al Cutting edge: 1,25-dihydroxyvitamin D3 is a direct inducer of antimicrobial peptide gene expression. J Immunol Baltim Md 1950. 2004;173: 2909–2912. 10.4049/jimmunol.173.5.2909 15322146

[8] Liu PT, Stenger S, Li H, Wenzel L, Tan BH, Krutzik SR, et al Toll-like receptor triggering of a vitamin D-mediated human antimicrobial response. Science. 2006;311: 1770–1773. 10.1126/science.1123933 16497887

[9] Schauber J, Dorschner RA, Coda AB, Büchau AS, Liu PT, Kiken D, et al Injury enhances TLR2 function and antimicrobial peptide expression through a vitamin D-dependent mechanism. J Clin Invest. 2007;117: 803–811. 10.1172/JCI30142 17290304 PMC1784003

[10] Li Z, Burns AR, Smith CW. Two waves of neutrophil emigration in response to corneal epithelial abrasion: distinct adhesion molecule requirements. Invest Ophthalmol Vis Sci. 2006;47: 1947–1955. 10.1167/iovs.05-1193 16639002

[11] Li Z, Rumbaut RE, Burns AR, Smith CW. Platelet response to corneal abrasion is necessary for acute inflammation and efficient re-epithelialization. Invest Ophthalmol Vis Sci. 2006;47: 4794–4802. 10.1167/iovs.06-0381 17065490

[12] Li Z, Burns AR, Rumbaut RE, Smith CW. gamma delta T cells are necessary for platelet and neutrophil accumulation in limbal vessels and efficient epithelial repair after corneal abrasion. Am J Pathol. 2007;171: 838–845. 10.2353/ajpath.2007.070008 17675580 PMC1959478

[13] Liu Q, Smith CW, Zhang W, Burns AR, Li Z. NK cells modulate the inflammatory response to corneal epithelial abrasion and thereby support wound healing. Am J Pathol. 2012;181: 452–462. 10.1016/j.ajpath.2012.04.010 22728064 PMC3409433

[14] Wilson SE, Mohan RR, Mohan RR, Ambrósio R, Hong J, Lee J. The corneal wound healing response: cytokine-mediated interaction of the epithelium, stroma, and inflammatory cells. Prog Retin Eye Res. 2001;20: 625–637. 11470453 10.1016/s1350-9462(01)00008-8

[15] Wilson SE, He YG, Weng J, Li Q, McDowall AW, Vital M, et al Epithelial injury induces keratocyte apoptosis: hypothesized role for the interleukin-1 system in the modulation of corneal tissue organization and wound healing. Exp Eye Res. 1996;62: 325–327. 10.1006/exer.1996.0038 8795451

[16] Yu F-SX, Yin J, Xu K, Huang J. Growth factors and corneal epithelial wound healing. Brain Res Bull. 2010;81: 229–235. 10.1016/j.brainresbull.2009.08.024 19733636 PMC3010187

[17] Lam FW, Burns AR, Smith CW, Rumbaut RE. Platelets enhance neutrophil transendothelial migration via P-selectin glycoprotein ligand-1. Am J Physiol Heart Circ Physiol. 2011;300: H468–475. 10.1152/ajpheart.00491.2010 21169400 PMC3044064

[18] Li Z, Burns AR, Smith CW. Lymphocyte function-associated antigen-1-dependent inhibition of corneal wound healing. Am J Pathol. 2006;169: 1590–1600. 10.2353/ajpath.2006.060415 17071583 PMC1780217

[19] Gao N, Yin J, Yoon GS, Mi Q-S, Yu F-SX. Dendritic cell-epithelium interplay is a determinant factor for corneal epithelial wound repair. Am J Pathol. 2011;179: 2243–2253. 10.1016/j.ajpath.2011.07.050 21924232 PMC3204011

[20] Murphy CJ, Marfurt CF, McDermott A, Bentley E, Abrams GA, Reid TW, et al Spontaneous chronic corneal epithelial defects (SCCED) in dogs: clinical features, innervation, and effect of topical SP, with or without IGF-1. Invest Ophthalmol Vis Sci. 2001;42: 2252–2261. 11527938

[21] Kubilus JK, Linsenmayer TF. Developmental corneal innervation: interactions between nerves and specialized apical corneal epithelial cells. Invest Ophthalmol Vis Sci. 2010;51: 782–789. 10.1167/iovs.09-3942 19741242 PMC2868469

[22] Marfurt CF, Ellis LC. Immunohistochemical localization of tyrosine hydroxylase in corneal nerves. J Comp Neurol. 1993;336: 517–531. 10.1002/cne.903360405 7902365

[23] Müller LJ, Marfurt CF, Kruse F, Tervo TMT. Corneal nerves: structure, contents and function. Exp Eye Res. 2003;76: 521–542. 12697417 10.1016/s0014-4835(03)00050-2

[24] Yu CQ, Zhang M, Matis KI, Kim C, Rosenblatt MI. Vascular endothelial growth factor mediates corneal nerve repair. Invest Ophthalmol Vis Sci. 2008;49: 3870–3878. 10.1167/iovs.07-1418 18487369 PMC3725763

[25] Shaheen BS, Bakir M, Jain S. Corneal nerves in health and disease. Surv Ophthalmol. 2014;59: 263–285. 10.1016/j.survophthal.2013.09.002 24461367 PMC4004679

[26] He J, Bazan NG, Bazan HEP. Mapping the entire human corneal nerve architecture. Exp Eye Res. 2010;91: 513–523. 10.1016/j.exer.2010.07.007 20650270 PMC2939211

[27] Li Z, Burns AR, Han L, Rumbaut RE, Smith CW. IL-17 and VEGF are necessary for efficient corneal nerve regeneration. Am J Pathol. 2011;178: 1106–1116. 10.1016/j.ajpath.2010.12.001 21356362 PMC3069816

[28] Wang SB, Hu KM, Seamon KJ, Mani V, Chen Y, Gronert K. Estrogen negatively regulates epithelial wound healing and protective lipid mediator circuits in the cornea. FASEB J Off Publ Fed Am Soc Exp Biol. 2012;26: 1506–1516. 10.1096/fj.11-198036 PMC3316908 22186873

[29] Biggs DSC. 3D deconvolution microscopy. Curr Protoc Cytom Editor Board J Paul Robinson Manag Ed Al. 2010;Chapter 12: Unit 12.19.1–20. 10.1002/0471142956.cy1219s52 20373494

[30] Xue M-L, Thakur A, Willcox M. Macrophage inflammatory protein-2 and vascular endothelial growth factor regulate corneal neovascularization induced by infection with Pseudomonas aeruginosa in mice. Immunol Cell Biol. 2002;80: 323–327. 10.1046/j.1440-1711.2002.01094.x 12121220

[31] Huang LC, Petkova TD, Reins RY, Proske RJ, McDermott AM. Multifunctional roles of human cathelicidin (LL-37) at the ocular surface. Invest Ophthalmol Vis Sci. 2006;47: 2369–2380. 10.1167/iovs.05-1649 16723446

[32] Carlson EC, Sun Y, Auletta J, Kao WW-Y, Liu C-Y, Perez VL, et al Regulation of corneal inflammation by neutrophil-dependent cleavage of keratan sulfate proteoglycans as a model for breakdown of the chemokine gradient. J Leukoc Biol. 2010;88: 517–522. 10.1189/jlb.0310134 20495072 PMC2924604

[33] Carlson EC, Lin M, Liu C-Y, Kao WW-Y, Perez VL, Pearlman E. Keratocan and lumican regulate neutrophil infiltration and corneal clarity in lipopolysaccharide-induced keratitis by direct interaction with CXCL1. J Biol Chem. 2007;282: 35502–35509. 10.1074/jbc.M705823200 17911102 PMC3909483

[34] Amano S, Rohan R, Kuroki M, Tolentino M, Adamis AP. Requirement for vascular endothelial growth factor in wound- and inflammation-related corneal neovascularization. Invest Ophthalmol Vis Sci. 1998;39: 18–22. 9430540

[35] Jung MK, Ha S, Huh SY, Park SB, Kim S, Yang Y, et al Hair-growth stimulation by conditioned medium from vitamin D3-activated preadipocytes in C57BL/6 mice. Life Sci. 2015;128: 39–46. 10.1016/j.lfs.2015.02.018 25748421

[36] Ren Z, Li W, Zhao Q, Ma L, Zhu J. The impact of 1,25-dihydroxy vitamin D3 on the expressions of vascular endothelial growth factor and transforming growth factor-β₁ in the retinas of rats with diabetes. Diabetes Res Clin Pract. 2012;98: 474–480. 10.1016/j.diabres.2012.09.028 23089551

[37] Yildirim B, Guler T, Akbulut M, Oztekin O, Sariiz G. 1-alpha,25-dihydroxyvitamin D3 regresses endometriotic implants in rats by inhibiting neovascularization and altering regulation of matrix metalloproteinase. Postgrad Med. 2014;126: 104–110. 10.3810/pgm.2014.01.2730 24393757

[38] Xue M-L, Zhu H, Thakur A, Willcox M. 1 alpha,25-Dihydroxyvitamin D3 inhibits pro-inflammatory cytokine and chemokine expression in human corneal epithelial cells colonized with Pseudomonas aeruginosa. Immunol Cell Biol. 2002;80: 340–345. 10.1046/j.1440-1711.80.4august.1.x 12121222

[39] Suzuki T, Sano Y, Sotozono C, Kinoshita S. Regulatory effects of 1alpha,25-dihydroxyvitamin D(3) on cytokine production by human corneal epithelial cells. Curr Eye Res. 2000;20: 127–130. 10617914

[40] Huang LC, Reins RY, Gallo RL, McDermott AM. Cathelicidin-deficient (Cnlp -/-) mice show increased susceptibility to Pseudomonas aeruginosa keratitis. Invest Ophthalmol Vis Sci. 2007;48: 4498–4508. 10.1167/iovs.07-0274 17898271 PMC4234056

[41] Byeseda SE, Burns AR, Dieffenbaugher S, Rumbaut RE, Smith CW, Li Z. ICAM-1 is necessary for epithelial recruitment of gammadelta T cells and efficient corneal wound healing. Am J Pathol. 2009;175: 571–579. 10.2353/ajpath.2009.090112 19608878 PMC2716957

[42] Suzuki K, Saito J, Yanai R, Yamada N, Chikama T, Seki K, et al Cell-matrix and cell-cell interactions during corneal epithelial wound healing. Prog Retin Eye Res. 2003;22: 113–133. 12604055 10.1016/s1350-9462(02)00042-3

[43] Terai K, Call MK, Liu H, Saika S, Liu C-Y, Hayashi Y, et al Crosstalk between TGF-β and MAPK Signaling during Corneal Wound Healing. Invest Ophthalmol Vis Sci. 2011;52: 8208–8215. 10.1167/iovs.11-8017 21917935 PMC3208026

[44] Sel S, Trau S, Paulsen F, Kalinski T, Stangl GI, Nass N. 1,25-dihydroxyvitamin D3 inhibits corneal wound healing in an ex-vivo mouse model. Graefes Arch Clin Exp Ophthalmol Albrecht Von Graefes Arch Klin Exp Ophthalmol. 2016; 10.1007/s00417-016-3267-4 26794222

[45] Elizondo RA, Yin Z, Lu X, Watsky MA. Effect of vitamin d receptor knockout on cornea epithelium wound healing and tight junctions. Invest Ophthalmol Vis Sci. 2014;55: 5245–5251. 10.1167/iovs.13-13553 25061117 PMC4142771

[46] Savill J. Apoptosis in resolution of inflammation. J Leukoc Biol. 1997;61: 375–380. 9103222 10.1002/jlb.61.4.375

[47] Weeres MA, Robien K, Ahn Y-O, Neulen M-L, Bergerson R, Miller JS, et al The effects of 1,25-dihydroxyvitamin D3 on in vitro human NK cell development from hematopoietic stem cells. J Immunol Baltim Md 1950. 2014;193: 3456–3462. 10.4049/jimmunol.1400698 PMC4363084 25149465

[48] Imanishi J, Kamiyama K, Iguchi I, Kita M, Sotozono C, Kinoshita S. Growth factors: importance in wound healing and maintenance of transparency of the cornea. Prog Retin Eye Res. 2000;19: 113–129. 10614683 10.1016/s1350-9462(99)00007-5

[49] Klenkler B, Sheardown H. Growth factors in the anterior segment: role in tissue maintenance, wound healing and ocular pathology. Exp Eye Res. 2004;79: 677–688. 10.1016/j.exer.2004.07.008 15500826

[50] Saika S. TGFbeta pathobiology in the eye. Lab Investig J Tech Methods Pathol. 2006;86: 106–115. 10.1038/labinvest.3700375 16341020

[51] Saika S, Okada Y, Miyamoto T, Yamanaka O, Ohnishi Y, Ooshima A, et al Role of p38 MAP kinase in regulation of cell migration and proliferation in healing corneal epithelium. Invest Ophthalmol Vis Sci. 2004;45: 100–109. 14691160 10.1167/iovs.03-0700

[52] Dekaris I, Zhu SN, Dana MR. TNF-alpha regulates corneal Langerhans cell migration. J Immunol Baltim Md 1950. 1999;162: 4235–4239. 10201952

[53] Hong JW, Liu JJ, Lee JS, Mohan RR, Mohan RR, Woods DJ, et al Proinflammatory chemokine induction in keratocytes and inflammatory cell infiltration into the cornea. Invest Ophthalmol Vis Sci. 2001;42: 2795–2803. 11687520

[54] Chen L, Meng Q, Kao W, Xia Y. IκB kinase β regulates epithelium migration during corneal wound healing. PloS One. 2011;6: e16132 10.1371/journal.pone.0016132 21264230 PMC3022035

[55] Carretero M, Escámez MJ, García M, Duarte B, Holguín A, Retamosa L, et al In vitro and in vivo wound healing-promoting activities of human cathelicidin LL-37. J Invest Dermatol. 2008;128: 223–236. 10.1038/sj.jid.5701043 17805349

[56] Gordon YJ, Huang LC, Romanowski EG, Yates KA, Proske RJ, McDermott AM. Human cathelicidin (LL-37), a multifunctional peptide, is expressed by ocular surface epithelia and has potent antibacterial and antiviral activity. Curr Eye Res. 2005;30: 385–394. 10.1080/02713680590934111 16020269 PMC1497871

[57] Steinstraesser L, Koehler T, Jacobsen F, Daigeler A, Goertz O, Langer S, et al Host defense peptides in wound healing. Mol Med Camb Mass. 2008;14: 528–537. 10.2119/2008-00002.Steinstraesser 18385817 PMC2277318

[58] Kai-Larsen Y, Agerberth B. The role of the multifunctional peptide LL-37 in host defense. Front Biosci J Virtual Libr. 2008;13: 3760–3767. 10.2741/2964 18508470

[59] McDermott AM. Defensins and other antimicrobial peptides at the ocular surface. Ocul Surf. 2004;2: 229–247. 17216098 10.1016/s1542-0124(12)70111-8 PMC1773007

[60] Huang LC, Jean D, Proske RJ, Reins RY, McDermott AM. Ocular surface expression and in vitro activity of antimicrobial peptides. Curr Eye Res. 2007;32: 595–609. 10.1080/02713680701446653 17852183 PMC2430515

[61] Agerberth B, Charo J, Werr J, Olsson B, Idali F, Lindbom L, et al The human antimicrobial and chemotactic peptides LL-37 and α-defensins are expressed by specific lymphocyte and monocyte populations. Blood. 2000;96: 3086–3093. 11049988

[62] Reins RY, McDermott AM. Vitamin D: Implications for ocular disease and therapeutic potential. Exp Eye Res. 2015;134: 101–110. 10.1016/j.exer.2015.02.019 25724179 PMC4426046

[63] Robertson DM, Li L, Fisher S, Pearce VP, Shay JW, Wright WE, et al Characterization of growth and differentiation in a telomerase-immortalized human corneal epithelial cell line. Invest Ophthalmol Vis Sci. 2005;46: 470–478. 10.1167/iovs.04-0528 15671271

