# Supplementary figures and images for: Effects of Topically Applied Vitamin D during Corneal Wound Healing
Source: PLoS One. 2016 Apr 1;11(4):e0152889. doi: 10.1371/journal.pone.0152889 (PMC4817982; doi:10.1371/journal.pone.0152889)

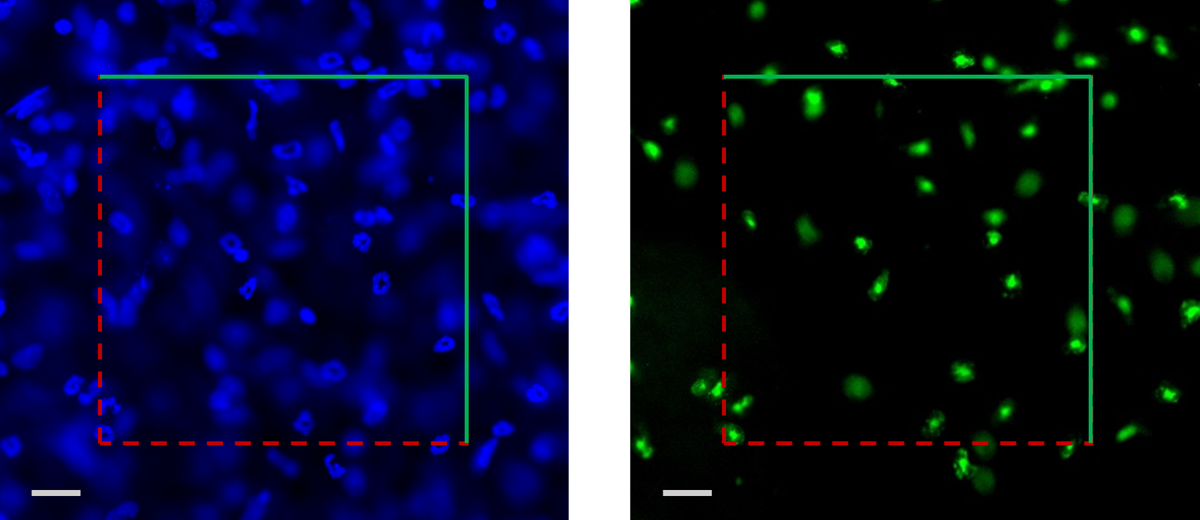

Supplement: S1 Fig — Morphometric counting frame with the accepted line in green and forbidden line in red dashes. Neutrophil “donut-shaped” nuclei were counted using DAPI (blue) and cross-checked with FITC-labeled Ly6G staining (green) throughout the z-stack in each image. Scale bar = 20μm. (TIF) [file pone.0152889.s001.tif]

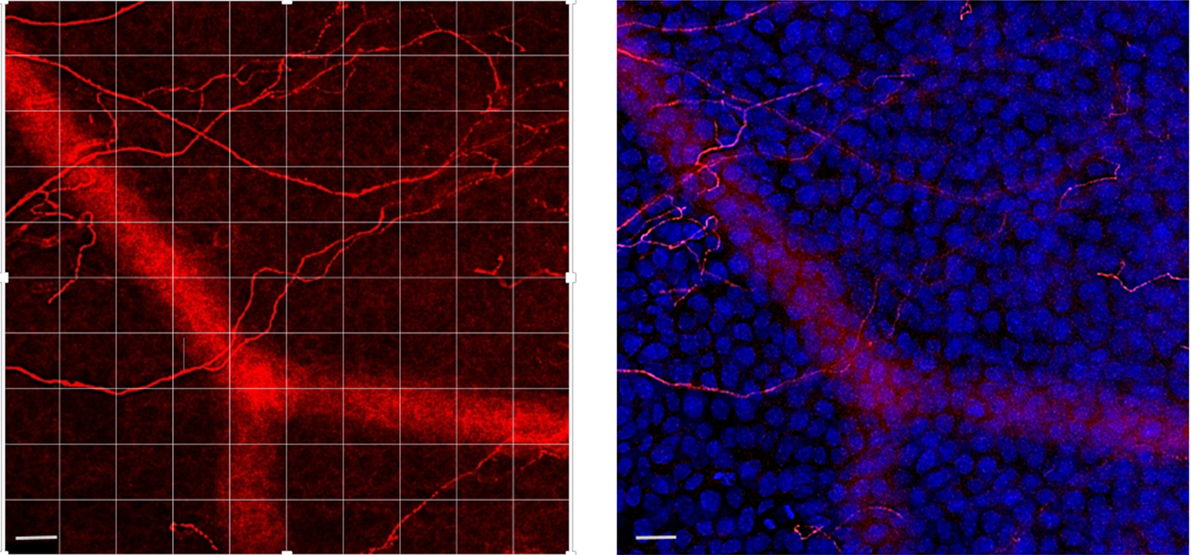

Supplement: S2 Fig — The left panel shows the 10x10 grid used to count subbasal epithelial nerves stained with NorthernLights™ NL557-conjugated anti-β-III tubulin (red). In this image, 57 out of 100 boxes contain a subbasal nerve fiber. A large stromal nerve can be seen beneath the plane of the thin subbasal nerves. On the right, basal epithelial cells are visible, stained with DAPI (blue). Scale bar = 20μm. (TIF) [file pone.0152889.s002.tif]

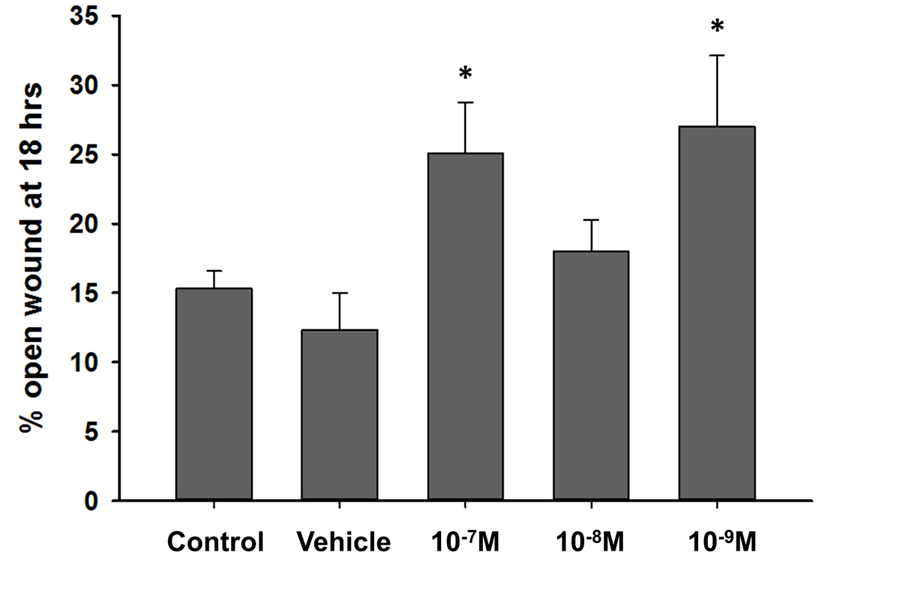

Supplement: S3 Fig — An initial wounding experiment was performed to test the effect of a range of vitamin D concentrations (10−7 to 10-9M) on corneal wound closure. Mice were imaged immediately after corneal wounding and at 18 hours post-wound with fluorescein staining to visualize wound area. Mice received topical vehicle (0.02% ethanol/PBS) or 1,253 (10−7, 10−8, or 10-9M) twice at the time of wounding and every 6 hours through 18 hours. Wound area remaining open was determined as a percentage of original wound area. Data represent mean ± SEM and were analyzed with one-way ANOVA and Bonferroni’s correction for multiple comparisons, p<*0.05 (n = 3 mice/group). (TIF) [file pone.0152889.s003.tif]

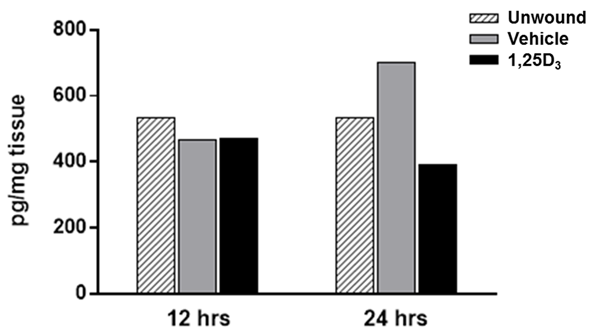

Supplement: S4 Fig — To determine vitamin D’s effect on pro-inflammatory cytokine expression following epithelial wounding, TNFα protein levels were determined in corneal homogenates 12 and 24 hours after wounding by ELISA. At 12 hours post-wounding, there was no change in TNFα protein with treatment. However, at 24 hours, vitamin D treatment decreased protein expression in corneal homogenates compared to vehicle (700pg/mg compared to 391pg/mg). Data represent 8 pooled corneas per group at each time point. Graphs are representative data from one experiment (n = 2, 24hrs; n = 1, 12hrs) showing the mean of duplicate values. (TIF) [file pone.0152889.s004.tif]

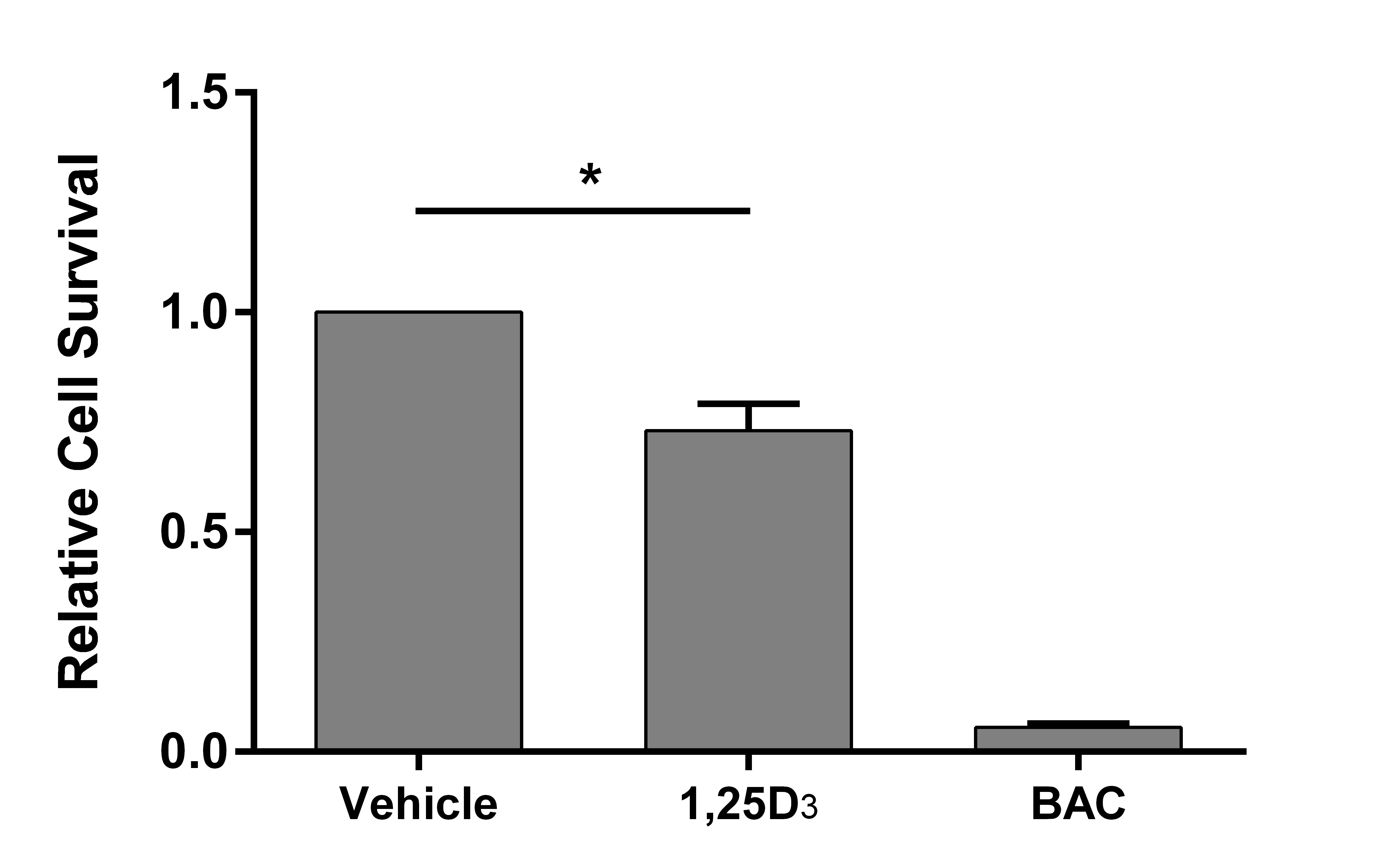

Supplement: S5 Fig — Human corneal epithelial cells (hTCEpi [63]) were plated in 96 well plates and treated with 1,25D3 (10-7M). Following 24 hours incubation, 0.5mg/ml MTT (3-(4, 5-dimethylthiazolyl-2)-2, 5-diphenyltetrazolium bromide) was added to each well and cells were incubated for an additional 2 hours at 37°C. Colorimetric changes were measured at wavelength 590 on a spectrophotometer and OD values normalized to untreated control cells. 0.02% benzalkonium chloride in PBS (BAC) was used as a positive control, indicating loss of cell viability. Data represent mean +/- SEM of 4 independent experiments. Statistical analysis was by Student’s t-test with * p = 0.022. (TIF) [file pone.0152889.s005.tif]
